# High density lipoproteins improve insulin sensitivity in high-fat diet-fed mice by suppressing hepatic inflammation[S]

**DOI:** 10.1194/jlr.M043281

**Published:** 2014-03

**Authors:** Kristine C. McGrath, Xiao Hong Li, Phillippa T. Whitworth, Robert Kasz, Joanne T. Tan, Susan V. McLennan, David S. Celermajer, Philip J. Barter, Kerry-Anne Rye, Alison K. Heather

**Affiliations:** * Faculty of Science, School of Medical and Molecular Biosciences, University of Technology-Sydney, Sydney, NSW, Australia; † Heart Research Institute, Newtown, NSW, Australia; § Department of Endocrinology, Dezhou People's Hospital, Shandong, China; ** Discipline of Medicine, University of Sydney, Sydney, NSW, Australia; †† Department of Cardiology, Royal Prince Alfred Hospital, Camperdown, NSW, Australia; and; §§ Faculty of Medicine, University of New South Wales, Randwick, NSW, Australia

**Keywords:** insulin resistance, apolipoprotein AI, cellular signalling

## Abstract

Obesity-induced liver inflammation can drive insulin resistance. HDL has anti-inflammatory properties, so we hypothesized that low levels of HDL would perpetuate inflammatory responses in the liver and that HDL treatment would suppress liver inflammation and insulin resistance. The aim of this study was to investigate the effects of lipid-free apoAI on hepatic inflammation and insulin resistance in mice. We also investigated apoAI as a component of reconstituted HDLs (rHDLs) in hepatocytes to confirm results we observed in vivo. To test our hypothesis, C57BL/6 mice were fed a high-fat diet (HFD) for 16 weeks and administered either saline or lipid-free apoAI. Injections of lipid-free apoAI twice a week for 2 or 4 weeks with lipid-free apoAI resulted in: *i*) improved insulin sensitivity associated with decreased systemic and hepatic inflammation; *ii*) suppression of hepatic mRNA expression for key transcriptional regulators of lipogenic gene expression; and *iii*) suppression of nuclear factor κB (NF-κB) activation. Human hepatoma HuH-7 cells exposed to rHDLs showed suppressed TNFα-induced NF-κB activation, correlating with decreased NF-κB target gene expression. We conclude that apoAI suppresses liver inflammation in HFD mice and improves insulin resistance via a mechanism that involves a downregulation of NF-κB activation.

Hepatic inflammation can induce insulin resistance via an imbalance in the secretion of pro-inflammatory cytokines, subsequent to activation of inflammatory/oxidative transcription factors (1). A key transcription factor that mediates the inflammatory response in hepatocytes is nuclear factor κB (NF-κB) (2, 3). Activated hepatic NF-κB alone can drive insulin resistance as evidenced by the finding that transgenic expression of inhibitor of nuclear factor κB kinase subunit β (IKK-β), which increases NF-κB activity, results in overt insulin resistance in mice fed a normal chow diet (4). Conversely, when heterozygous IKK-β^+/−^ mice expressing low levels of NF-κB are fed a high-fat diet (HFD), or are crossed with *ob/ob* mice, they do not develop insulin resistance (5). Moreover, genetic manipulation to inhibit hepatic NF-κB activity directly protects against insulin resistance in response to a HFD in mice (4). Such findings provide strong evidence that the liver is a primary site of inflammatory action that causes insulin resistance, and that NF-κB is a central pathogenic factor underlying inflammation-induced insulin resistance.

NF-κB activation increases the secretion of a number of pro-inflammatory cytokines, including interleukin (IL)-6, TNFα, and IL-1β (1). NF-κB activation involves a complex series of signaling events that begins with activation of the inhibitor κB (IκB) kinase complex that, in turn, phosphorylates IκB (6, 7). IκB is an inhibitor protein of NF-κB that binds to NF-κB, sequestering it in the cytoplasm (8). However, once phosphorylated, IκB is targeted for ubiquitination and subsequent degradation, leaving NF-κB free to translocate to the nucleus and initiate transcription of target genes (9).

High density lipoproteins (HDLs) have potent anti-inflammatory effects (10, 11). We have previously reported that pretreatment of human coronary artery endothelial cells with HDLs inhibits TNFα-induced NF-κB activation (12). In addition, injections of human apolipoprotein A-I (apoAI) (the major HDL protein) into rabbits inhibits vascular inflammation (10). HDL levels are reportedly low in subjects with insulin resistance (13), so this led us to question whether raising HDL might improve insulin resistance by targeting the heightened hepatic inflammatory state. We show that injections of lipid-free apoAI decrease both hepatic and systemic cytokine levels, suppress hepatic NF-κB activation, and improve insulin sensitivity in high-fat-fed C57BL/6 mice. Moreover, we demonstrate that apoAI-containing reconstituted HDLs (rHDLs) mediate their anti-inflammatory effects in cultured hepatocytes via suppression of the IκB kinase (IKK)/IκB/NF-κB signaling pathway.

## MATERIALS AND METHODS

### Preparation of lipid-free apoAI and discoidal rHDLs

HDLs were isolated from pooled human plasma (Gribbles Pathology, Adelaide, SA, Australia) by sequential ultracentrifugation in the 1.063–1.21 g/ml density range. Lipid-free apoAI was then isolated from HDL as previously described (14). Discoidal rHDLs containing apoAI complexed to 1-palmitoyl-2-linoleoyl-*sn*-glycero-3-phosphatidylcholine (PLPC) (Avanti Polar Lipids, Alabaster, AL) were prepared by the cholate dialysis method (15). The PLPC:apoAI molar ratio was 100:1. Immediately before use in experiments, lipid-free apoAI or rHDL preparations were dialyzed extensively against endotoxin-free phosphate buffered saline (PBS) (pH 7.4) (Sigma-Aldrich, Castle Hill, NSW, Australia).

### Animal model

Five-week-old C57BL/6 male mice purchased from Monash Animal Services (Monash University, VIC, Australia) were housed at the Heart Research Institute biological services facility (Sydney, NSW, Australia) and kept at a temperature of 21 ± 2°C on a 12:12 h light/dark cycle. All experiments were approved by the South Western Sydney Area Health Service Animal Welfare Committee. At 6 weeks of age, the mice were randomized into four groups: *1*) Control mice (n = 10) were fed a standard chow diet (StD) [14.3 MJ/kg; 65% of energy from carbohydrates (wheat, barley, lupins); 12% from fat (mixed vegetable oils, canola oil); 23% from protein (soya meal, fish meal)] (Specialty Feeds, Glen Forrest, WA, Australia). *2*) Mice (n = 10) were fed a HFD [19.4 MJ/kg; 43% of energy from carbohydrates (sucrose, cellulose, wheat starch); 40% from fat-clarified butter (Ghee), cholesterol; 17% from protein (casein)] (SF00-219, Specialty Feeds) ad libitum for 16 weeks total. At 18 weeks of age and 13 weeks of HFD, this subset of mice received endotoxin-free PBS (vehicle control) by tail vein injection twice weekly for the last 4 weeks of the diet. *3*) Mice (n = 10) were fed a HFD ad libitum for 16 weeks total. At 18 weeks of age and 13 weeks of HFD, this subset of mice received apoAI by tail vein injection twice weekly for the last 4 weeks of the diet. *4*) Mice (n = 10) were fed a HFD ad libitum for 16 weeks total. At 20 weeks of age and 15 weeks of HFD, this subset of mice received apoAI by tail vein injection twice weekly for the last 2 weeks of the diet. ApoA-I was administered at a dose of 8 mg/kg. Animals were euthanized at 24 h after their final injection by right atrium exsanguination after methoxyflurane anesthesia (Medical Developments International, Springvale, VIC, Australia). Blood samples were centrifuged (1,000 *g* for 10 min) and the subsequent serum fraction collected and stored at −20°C. Liver tissue was rapidly excised, snap-frozen in liquid nitrogen, and stored at −80°C.

### Glucose tolerance, insulin tolerance, and pyruvate challenge tests; fasting serum triglyceride, cytokine, and insulin measurements

At the end of the study, an intraperitoneal glucose tolerance test (IPGTT), an intraperitoneal insulin tolerance test (IPITT), and a pyruvate challenge test were performed in overnight-fasted mice. Blood samples were obtained from the tail tip at the indicated times and glucose levels were measured using a glucometer (Accu-Chek, Roche Diagnostics, Castle Hill, NSW, Australia). The doses used during these tests were glucose at 1 g/kg body weight, insulin at 0.75 IU/kg body weight, and pyruvate at 2 g/kg body weight for the IPGTT, IPITT, and pyruvate challenge test, respectively. Serum triglyceride levels were measured with triglyceride reagent (Roche Diagnostics). Serum cytokine levels were determined using a mouse BioPlex kit (Bio-Rad, Hercules, CA). Insulin levels were measured using an enzyme-linked immunosorbent assay (Crystal Chem, Downers Grove, IL). The homeostatic model assessment of insulin resistance (HOMA-IR) was determined for each mouse (4).

### Intrahepatic neutral lipid accumulation assay

The level of neutral lipids (triglyceride plus cholesterol esters) accumulated in the liver was determined by measurement of Oil Red-O of tissue extracts by quantitative assay (16). Briefly, frozen liver tissue (100 mg) was homogenized and incubated with an Oil Red-O solution (0.15% Oil Red-O, 0.4% dextrin) for 60 min. The samples were washed with 60% isopropanol to remove excess dye and the dye incorporated into lipid was then extracted with 99% isopropanol and quantified by measurement of absorbance at 520 nm (16).

### Cell culture

A human hepatoma cell line (HuH-7) (Health Science Research Resources Bank, Osaka, Japan) was cultured in DMEM/F12 medium (Sigma-Aldrich) with 10% FBS at 37°C in 5% CO_2_. Unless otherwise stated, HuH-7 cells were preincubated for 16 h with rHDLs (final apoAI concentration, 16 μmol/l or 0.45 mg/ml), PBS (control), sodium salicylate (5 mmol/l) (Sigma-Aldrich), or the IKK inhibitor, wedelolactone (8 mmol/l) (Calbiochem, Gibbstown, NJ) and then stimulated with TNFα (1 ng/ml) for 24 h. For some cells, after the 16 h incubation, the rHDL-containing medium was removed and cells were washed twice with PBS before fresh medium supplemented with TNFα (1 ng/ml) was added for 24 h. NonTNFα-stimulated PBS-treated cells acted as controls.

### Cholesterol depletion and repletion

Cholesterol depletion was performed by incubating HuH-7 cells with 1.5% methyl-β-cyclodextrin for 1 h at 37°C. To perform cholesterol repletion, cholesterol (0.4 mg/ml) was mixed by vortexing with methyl-β-cyclodextrin (10%) at a 1:20 ratio at 40°C. Following incubation of HuH-7 cells with rHDLs for 16 h, cholesterol repletion was performed by the addition of the cholesterol/cyclodextrin solution diluted at 1:25 for another hour.

### Lactate dehydrogenase cell viability assay

HuH-7 cells were incubated for 40 h with rHDLs (final apoAI concentration, 16 μmol/l or 0.45 mg/ml), PBS (control), or sodium salicylate (5 mmol/l, Sigma-Aldrich). Following incubation, cell media were collected and stored on ice. The cells were then washed with PBS and lysed in 1 ml of water for 20 min at 4°C. After centrifugation (1,000 *g*, 5 min) to pellet and remove cell debris, 10 μl cell lysate or cell media were incubated with 200 μl of 0.15 mg/ml NADH and 2.5 mmol/l sodium pyruvate PBS working reagent. The absorbance at 340 nm was determined at 5 min intervals for 35 min (Tecan Sunrise; Tecan Group Ltd., Mannedorf, Switzerland). Viability was calculated from the relative activity of lactate dehydrogenase measured for the media versus total activity (17).

### Transient cell transfections and luciferase measurements

HuH-7 cells were transfected with an NF-κB-luciferase reporter vector (Promega Corporation, Madison, WI) together with a transfection control plasmid, pRL-TK (Promega), using Effectene (Qiagen, Hilden, Germany) (18). Transfected cells were preincubated with rHDLs (final apoAI concentration, 16 μmol/l or 0.45 mg/ml) then stimulated with 1 ng/ml TNFα (rHDL+TNFα). A subset of transfected cells was preincubated with rHDLs, but the rHDLs were removed from the culture media prior to activation with TNFα (rHDL//TNFα). After treatment, cells were washed with PBS and then lysed with passive lysis buffer (Promega). Samples were collected, centrifuged to remove cell debris, and then assayed for luciferase and Renilla activity using the Dual-Luciferase reporter system (Promega). Measurements were obtained using the Fluoroskan Ascent FL luminometer (Thermo Labsystems, Waltham, MA).

### IKK assay

HuH-7 cells were pretreated with rHDLs (final apoAI concentration, 16 μmol/l or 0.45 mg/ml) or PBS, and then stimulated with TNFα (1 ng/ml) for 15 min. The cells were lysed in RIPA lysis buffer containing 1% Nonidet P-40, 0.1% SDS, 0.5% deoxycholate, 150 mmol/l NaCl, 50 mmol/l Tris (pH 8), and a protease inhibitor cocktail (Sigma-Aldrich). The protein lysate (10 μg) was incubated with 2 mmol/l ATP and 10 μg IKK substrate peptide (Millipore, Billerica, MA) in reaction buffer (8 mmol/l MOPS (pH 7), 0.2 mmol/l EDTA-Na_2_) at room temperature for 90 min. Kinase-Glo reagent (50 μl; Promega) was then added to the reaction mixture, incubated at room temperature for 10 min, and the luminescent signal was measured on a Fluoroskan Ascent FL (Thermo Labsystems).

### IκB assay

Whole cell protein lysate was extracted from HuH-7 cells in RIPA lysis buffer as described for the IKK assay. The protein lysate (100 mg) was assayed for IκBα levels using the PathScan phospho-IκBα and total IκBα enzyme-linked immunosorbent assay (Cell Signaling Technology, Danvers, MA).

### NF-κB nuclear translocation assay

Nuclear proteins were extracted from HuH-7 cells or liver samples using the NucBuster protein extraction kit (Merck and Co., Whitehouse Station, NJ). Nuclear proteins (100 μg) were assayed using the NF-κB NoShift transcription factor assay kit (Merck and Co.).

### Human NF-κB target gene array analysis

Total RNA was isolated from HuH-7 cells using TRI reagent (Sigma-Aldrich). Biotin-labeled cDNA probes were prepared from 10 μg of total RNA using AMV reverse transcriptase (Promega) and biotin dUTP. The cDNA probes were then hybridized to the TranSignal NF-κB target gene array (Panomics, Santa Clara, CA). Direct chemiluminescence imaging was performed using the ChemiDoc XRS (Bio-Rad) imaging system. Quantity One software (Bio-Rad) was used for pairwise comparative gene expression after signal intensities were converted to a ratio adjusted for background and reference gene expression. The array reproducibility was verified by RT-quantitative (q)PCR for genes of interest.

### Isolation of total mRNA and analysis by RT-qPCR

Total RNA was extracted from HuH-7 cells or liver samples using TRI reagent (Sigma-Aldrich) and the concentration normalized to 100 ng/μl using the SYBR Green II assay (Molecular Probes, Invitrogen, Melbourne, Australia). RNA integrity was determined with the Experion system (Bio-Rad). cDNA was reverse transcribed from total RNA (100 ng) using iSCRIPT (Bio-Rad). Gene expression (see supplementary Table I for primer sequences) was amplified by PCR in reaction mixtures containing 12 pmol primers and iQ SYBR Green Supermix. Amplification was performed in a Bio-Rad iQ5 thermocycler using the following protocol: 95°C for 30 s, 60°C for 30 s, and 72°C for 30 s. Relative change in mRNA gene expression was determined by the ΔΔCT approach (19), using β-2-microglobulin (*β2M*) levels as the reference gene for human samples or transcription factor IID (*Tbp*) for mouse samples.

### Statistical analysis

Data are expressed as mean ± SEM. Significant differences in treatments were determined by one-way ANOVA with Bonferroni's post hoc test analysis. PRISM software was used for analysis. Significance was set at a two-tailed *P* value <0.05.

## RESULTS

### ApoAI improves glucose tolerance, insulin sensitivity, and hepatic glucose metabolism in HFD-fed mice

**Fig. 1.**
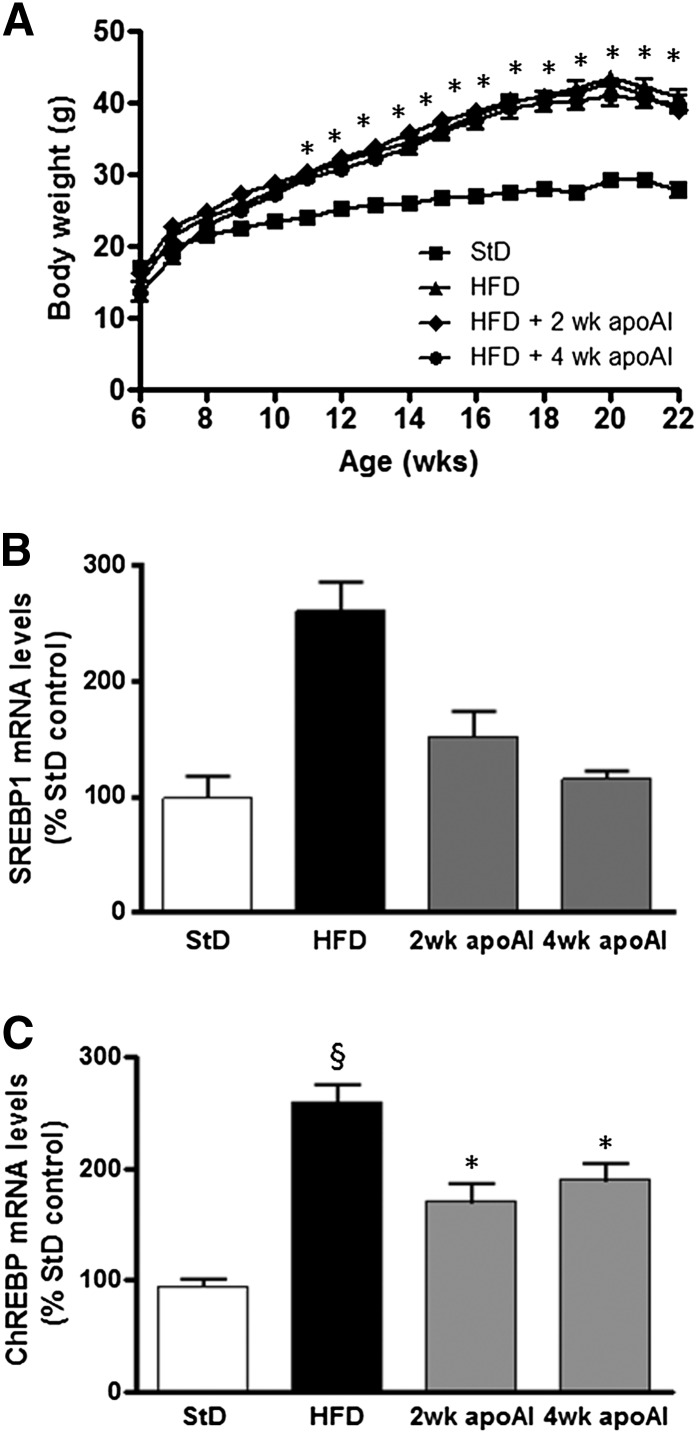
Body weights of C57BL/6 mice and hepatic mRNA levels of genes involved in fat synthesis. A: Beginning at 6 weeks of age, C57BL/6 mice were fed a StD or a HFD for 16 weeks. The HFD group was subdivided: HFD, was administered endotoxin-free PBS for the last 4 weeks of the diet; HFD + 2 weeks (wk) apoAI, was administered apoAI for the last 2 weeks of the diet; and HFD + 4 weeks (wk) apoAI, was administered apoAI for the last 4 weeks of the diet. Body weight was monitored once weekly (n = 10 for each treatment group). *P < 0.001 versus StD. *SREBP-1* (B) and *ChREBP* (C) mRNA levels were measured by qPCR. mRNA levels were normalized to transcription factor IID (*Tbp*). Results are mean ± SEM (n = 8–10). §*P <* 0.05 versus StD; *P < 0.05 versus HFD.

HFD-fed C57BL/6 mice had a 14–15 g (*P* < 0.05) increase in body weight compared with StD-fed mice over the 16 week study period (**Fig. 1A**). The increase in body weight was associated with increased serum triglyceride, hepatic neutral lipid (triglyceride plus cholesterol esters) levels (supplementary Fig. IIIA, B; *P* < 0.01 for both), and key transcriptional regulators of lipogenic gene expression in the liver sterol regulatory element binding protein 1 (SREBP-1) and carbohydrate responsive element binding protein (ChREBP) (*P* < 0.001; Fig. 1B, C). The HFD-induced increase in body weight was not affected by the apoAI treatments; however, it suppressed the HFD-induced increase in serum triglyceride, hepatic neutral lipid (triglyceride plus cholesterol esters) levels, *SREBP-1*, and *ChREBP* levels (supplementary Fig. IIIA, B and Fig. 1B, C, respectively).

**Fig. 2.**
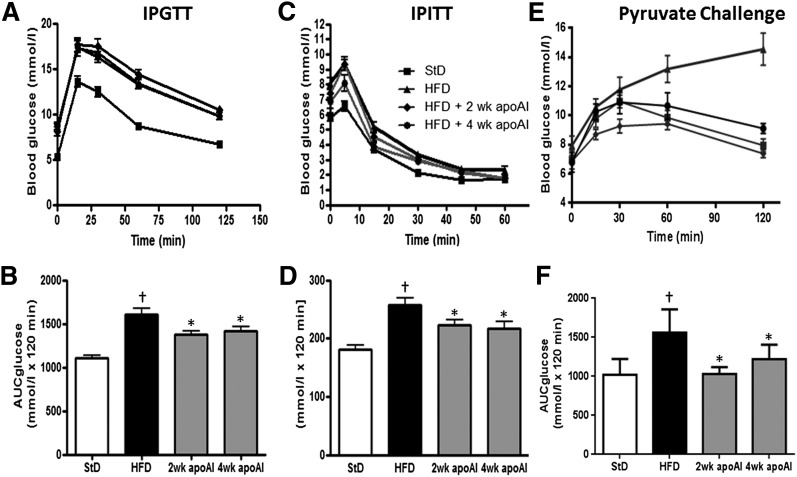
Plasma glucose concentrations during metabolic assays in C57BL/6 mice fed a StD or a HFD and treated with apoAI. IPGTT (glucose 1 g/kg) (A) and area under the curve for glucose (AUC glucose) (B) were calculated using the trapezoid rule. IPITT (insulin 0.75 IU/kg) (C) and AUC glucose (D). Pyruvate (pyruvate 2 g/kg) challenge test (E) and AUC glucose (F). Results are mean ± SEM (n = 8–10). †*P* < 0.05 versus StD; *P < 0.05 versus HFD.

Insulin resistance, as measured by the HOMA-IR (1), was 12-fold higher in the HFD-fed C57BL/6 mice than in the control animals (supplementary Fig. I). Both 2 and 4 weeks of treatment with apoAI reduced the HOMA-IR in the HFD-fed animals by 2.81 ± 0.97-fold and 2.85 ± 0.4-fold, respectively (*P* < 0.05 for both). HFD-fed mice displayed glucose intolerance, with levels of both fasting insulin (supplementary Fig. IIA, *P* < 0.05) and glucose (supplementary Fig. IIB, *P* < 0.05) being increased by 3.2 ± 0.5-fold and 1.44 ± 0.7-fold, respectively. Treatment with apoAI for 2 and 4 weeks significantly reduced fasting insulin and glucose levels. These results were consistent with the apoAI treatment improving IPGTT and IPITT (**Fig. 2A–D**). ApoAI treatment also improved hepatic glucose metabolism as determined by the pyruvate challenge assay (Fig. 2E, F). This was associated with an apoAI-induced suppression of mRNA levels encoding the rate-limiting enzymes in gluconeogenesis, phosphoenolpyruvate carboxykinase (PEPCK) and glucose-6-phosphatase (G6Pase) (**Table 1**).

**TABLE 1.** Hepatic mRNA gene expression

Encoded Protein	HFD/StD	Two Week apoAI/HFD	Four Week apoAI/HFD
PEPCK	1.95 ± 1.94↑*^a^*	0.43 ± 0.57↓*^a^*	0.45 ± 0.70↓*^a^*
G6Pase	1.76 ± 3.10↑*^a^*	0.54 ± 0.84↓*^a^*	0.41 ± 0.53↓*^b^*
TNFα	1.65 ± 4.00↑*^a^*	0.11 ± 0.10↓*^b^*	0.22 ± 0.17↓*^b^*
IL-6	1.33 ± 2.20↑*^a^*	0.49 ± 1.17↓*^b^*	0.30 ± 0.70↓*^b^*
IFN-γ	4.14 ± 3.10↑*^b^*	0.70 ± 0.66↓*^a^*	0.61 ± 1.00↓*^b^*
IL-1β	4.96 ± 6.11↑*^a^*	0.42 ± 0.25↓*^b^*	0.24 ± 0.19↓*^b^*
SAA1	21.06 ± 8.38↑*^b^*	0.51 ± 0.36↓*^a^*	0.25 ± 0.28↓*^b^*
CD68	4.43 ± 1.76↑*^b^*	0.64 ± 1.89↓*^a^*	0.58 ± 1.06↓*^a^*
F4/80	7.02 ± 5.87↑*^b^*	0.63 ± 1.46↓*^a^*	0.58 ± 0.99↓*^a^*

Beginning at 6 weeks of age, C57BL/6 mice were fed a StD or a HFD for 16 weeks. The HFD group was divided into three groups and administered apoAI (8 mg/kg) for 4 weeks, 2 weeks, or endotoxin-free PBS (HFD). Total RNA was isolated from liver tissue and mRNA levels measured by RT-qPCR. All cytokine mRNA levels were normalized to transcription factor IID (Tbp). RT-qPCR data represent fold increase over StD or HFD. Results are mean ± SEM (n = 8–10 animals for each treatment group).

a *P* < 0.05.

b *P* < 0.01.

### Cytokine expression following apoAI treatments

**Fig. 3.**
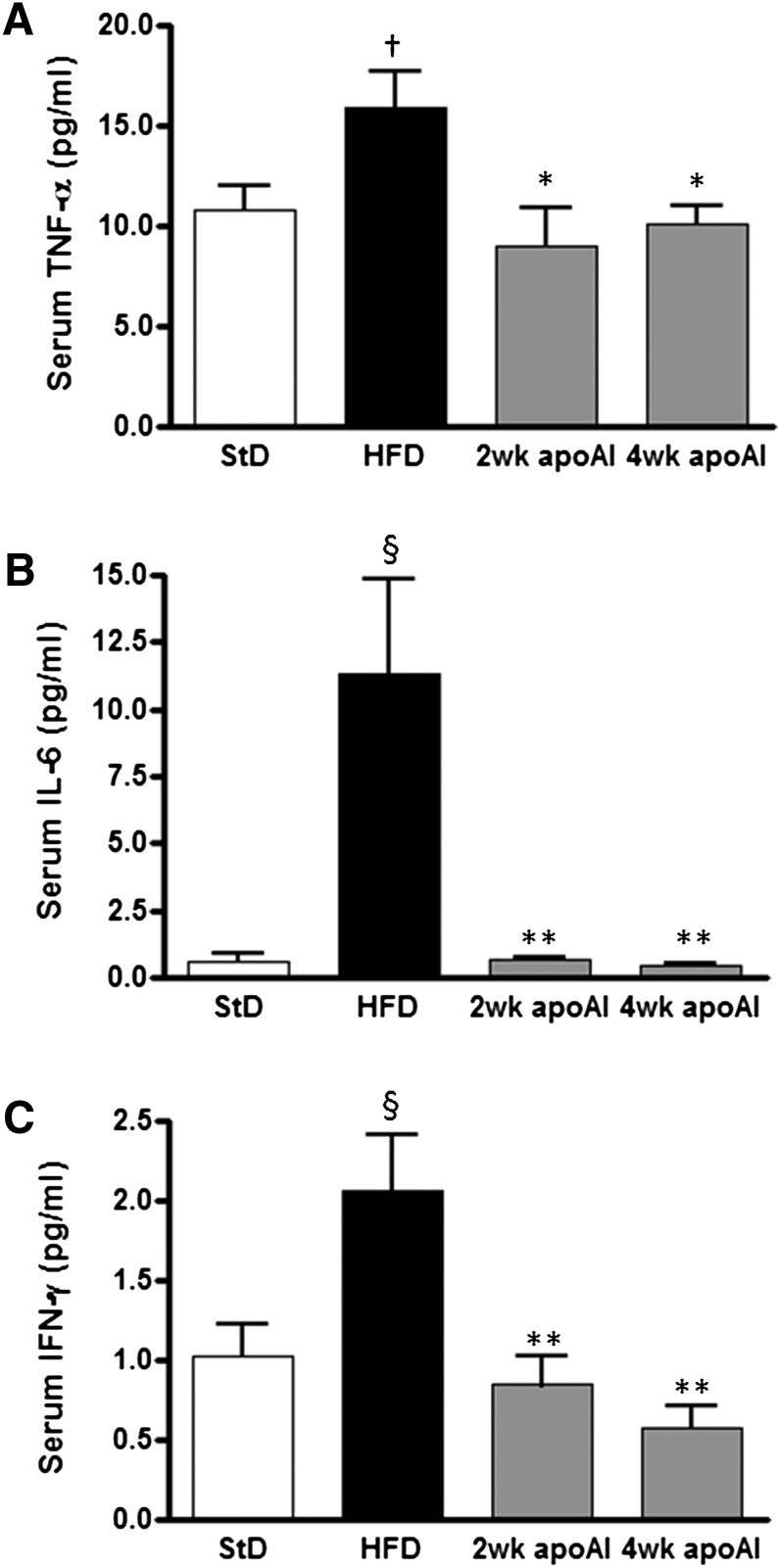
Serum cytokine levels in C57BL/6 mice treated with apoA-I. C57BL/6 mice were fed a StD or a HFD and treated with apoAI as described in the Materials and Methods. Circulating levels of TNFα (A), IL-6 (B), and IFN-γ (C) were determined using the BioPlex kit (Bio-Rad). Results are mean ± SEM (n = 8–10). †*P* < 0.05 versus StD; §*P* < 0.001 versus StD; *P < 0.05 versus HFD; **P < 0.01 versus HFD.

Serum cytokine levels were measured to assess systemic inflammation. HFD-fed mice had elevated serum concentrations of TNFα (*P* < 0.05), IL-6 (*P* < 0.001), and IFN-γ (*P* < 0.001) compared with StD-fed mice (**Fig. 3**). ApoAI treatment for both 2 and 4 weeks reduced serum levels of IL-6 by 94 ± 0.13% (*P <* 0.01) and 96 ± 9% (*P* < 0.01), respectively. Similarly, the apoAI treatments significantly reduced serum levels of IFN-γ and TNFα by 36–58 ± 9–19% (*P* < 0.05).

Table 1 shows that treatment with apoAI significantly decreased TNFα, IL-6, IFN-γ, IL-1β, and serum amyloid A1 (SAA1) hepatic mRNA levels in the HFD-fed mice. Mice consuming the HFD had an increased number of macrophages (Kupffer cells) in the liver as demonstrated by an increase in mRNA levels of the macrophage-specific genes *CD68* and *F4/80*. Treatment of the HFD mice with apoAI for 2 or 4 weeks decreased the hepatic expression of both *CD68* and *F4/80* (Table 1).

### ApoAI suppressed activated NF-κB levels

**Fig. 4.**
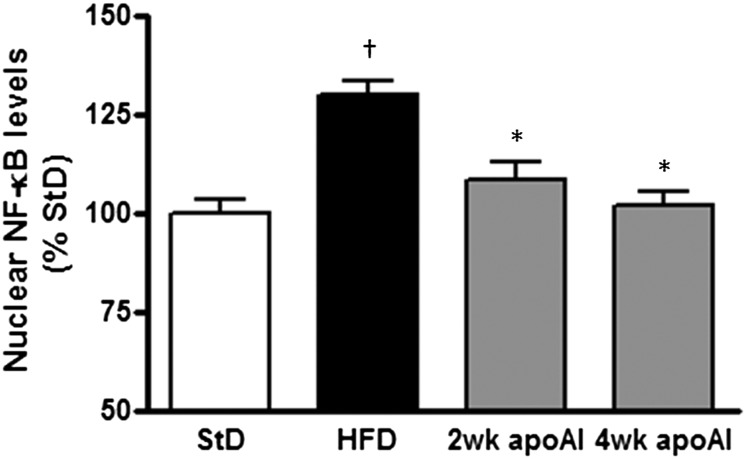
Administration of apoAI decreases hepatic NF-κB activity. C57BL/6 mice were fed a StD or a HFD and treated with apoAI as described in the Materials and Methods. Hepatic NF-κB activity was measured by an oligonucleotide specific for the NF-κB consensus DNA sequence. Results are mean ± SEM (n = 8–10). †*P* < 0.05 versus StD; *P < 0.05 versus HFD.

NF-κB is the key regulator of *TNFα*, *IL-6*, *IFN-γ*, *IL-1β*, and *SAA1* gene expression in the liver (20, 21). As shown in **Fig. 4**, the HFD-fed mice had a 30 ± 3% (*P* < 0.05) increase in hepatic nuclear NF-κB levels relative to levels in the StD-fed mice. Treatment with apoAI decreased NF-κB levels and, in the animals treated with apoAI for 4 weeks, the NF-κB nuclear levels were returned to those observed in the StD-fed mice.

We have previously shown that apoAI as a component of discoidal rHDLs suppressed NF-κB activation through increased expression of DHCR24, and that this enzyme is at least in part responsible for mediating the anti-inflammatory effects of rHDLs in human coronary aortic endothelial cells (12). Supplementary Fig. IV shows there was no change in hepatic DHCR24 mRNA expression in HFD-fed mice treated with apoAI for 2 or 4 weeks.

### rHDLs suppressed classical NF-κB signaling in cultured human hepatocytes

**Fig. 5.**
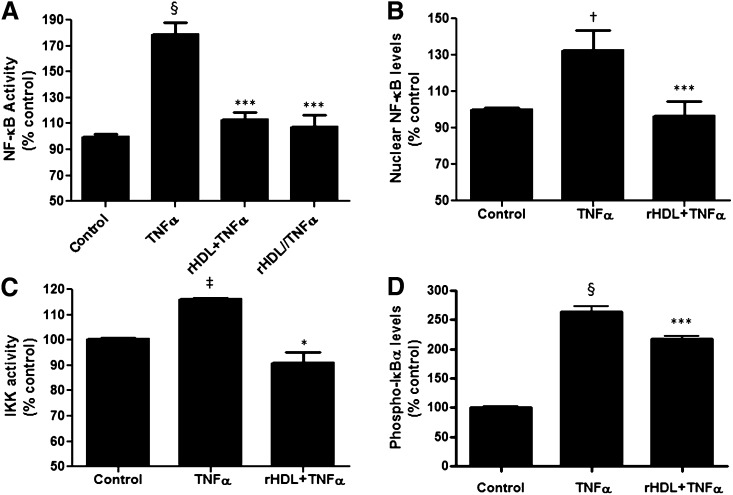
rHDLs suppress TNFα-activated signaling through the classical NF-κB pathway. A: HuH-7 cells transfected with an NF-κB-luciferase reporter vector. Transfected cells were treated with PBS (control), TNFα preincubated with rHDLs (final apoAI concentration 16 μmol/l or 0.45 mg/ml) then stimulated with TNFα (rHDL+TNFα), or preincubated with rHDLs for 16 h and the rHDLs removed from the culture media prior to activation with TNFα (rHDL//TNFα). Cells were then harvested, lysed, and the cell lysates were assayed for luciferase activity. Results are expressed as mean ± SEM (n = 5). §*P* < 0.001 versus control; ***P < 0.001 versus TNFα. B: rHDLs suppress TNFα-induced translocation of NF-κB in hepatocytes. HuH-7 cells were treated with PBS (control), TNFα, or preincubated with rHDLs (final apoAI concentration 16 μmol/l or 0.45 mg/ml) then stimulated with TNFα (rHDL+TNFα). Nuclear proteins were extracted and NF-κB levels measured. Results are expressed as mean ± SEM (n = 5). †*P* < 0.05 versus control; ***P < 0.001 versus TNFα. C: rHDLs suppress TNFα-activated IKK activity in hepatocytes. HuH-7 cells were pretreated with rHDLs (final apoAI concentration 16 μmol/l or 0.45 mg/ml) or PBS (control) for 16 h then exposed to TNFα for 15 min. Results are expressed as mean ± SEM (n = 5). ‡*P* < 0.01 versus control; *P < 0.05 versus TNFα. D: rHDLs prevent degradation of IκBα in hepatocytes. HuH-7 cells were preincubated for 16 h with rHDLs (final apoAI concentration 16 μmol/l or 0.45 mg/ml) or PBS (control) then stimulated with TNFα for 24 h. Protein lysates were extracted and the level of phosphorylated IκBα measured by ELISA. Results are expressed as mean ± SEM (n = 5). §*P* < 0.001 versus control; ***P < 0.001 versus TNFα.

The human transformed liver cell line (HuH-7) was transfected with an NF-κB-luciferase reporter vector, and then preincubated for 16 h with discoidal rHDLs (final apoAI concentration, 16 μmol/l or 0.45 mg/ml). After exposure to rHDLs, the HuH-7 cells were stimulated with TNFα for 5 h to induce inflammation. Cells pretreated with PBS alone before being activated with TNFα acted as positive controls. **Figure 5A** shows that exposure to TNFα increased NF-κB activation by 80 ± 8% (*P <* 0.0001), an effect that was abrogated in the cells pretreated with rHDLs (rHDL+TNFα). The effect of rHDLs was not due to blockade of the TNFα receptor, as the effects of the rHDLs were sustained even if the rHDLs were removed from the culture media prior to TNFα stimulation (rHDL//TNFα). Using a NF-κB nuclear translocation assay, Fig. 5B confirms the reporter assay results, with pretreatment with rHDLs decreasing TNFα-induced nuclear NF-κB levels.

We next examined whether rHDL (final apoAI concentration, 16 μmol/l or 0.45 mg/ml) treatment decreased IKK activation, thereby stopping IκBα degradation. As shown, TNFα increased IKK activity by 16 ± 0.7% (*P* < 0.01; Fig. 5C), this effect was abrogated by rHDL treatment (*P <* 0.05). Treatment with the rHDLs also effectively blocked IκBα phosphorylation as measured by an ELISA for phosphorylated-IκBα versus total IκBα (Fig. 5D).

### rHDL anti-inflammatory effects are not via cholesterol mobilization

**Fig. 6.**
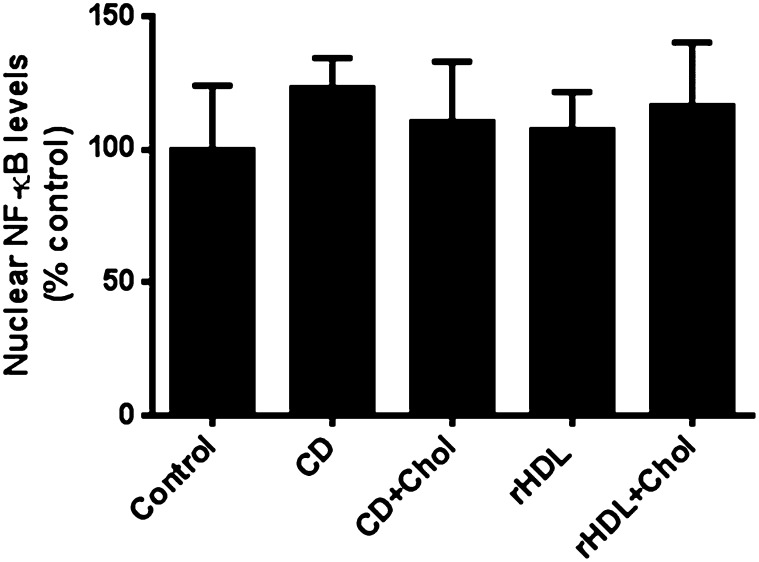
Cholesterol depletion/repletion has no effect on the activation of NF-κB. Cholesterol depletion was performed on HuH-7 cells by incubation with 1.5% cyclodextrin (CD) for 1 h. Cholesterol repletion was performed by the addition of CD plus cholesterol (CD+Chol) for an additional hour. To assess the effects of rHDL on cholesterol mobilization, cells were treated with rHDL (rHDL) (final apoAI concentration 16 μmol/l or 0.45 mg/ml) for 16 h followed by the addition of CD plus cholesterol (rHDL+Chol) for an additional hour. Following incubations, nuclear proteins were extracted and NF-κB levels measured. Results are expressed as mean ± SEM (n = 3).

The anti-inflammatory effects of rHDLs may be due to rHDL-mediated cholesterol mobilization from the plasma membrane. **Figure 6** shows cholesterol depletion by treatment with 1.5% methyl-β-cyclodextrin for 1 h or cholesterol repletion with a cholesterol/cyclodextrin mixture (final concentration of cholesterol, 16 μg/ml) for another hour did not significantly affect the activation of NF-κB compared with the control. Similarly, incubation of HuH-7 cells with rHDLs for 16 h or rHDLs for 16 h followed by cholesterol repletion for another hour with the cholesterol/cyclodextrin mixture also had no effect on the activation of NF-κB as measured by a NF-κB nuclear translocation assay.

### rHDLs are more effective than 5 mmol/l salicylate at inhibiting TNFα-induced NF-κB activity

**Fig. 7.**
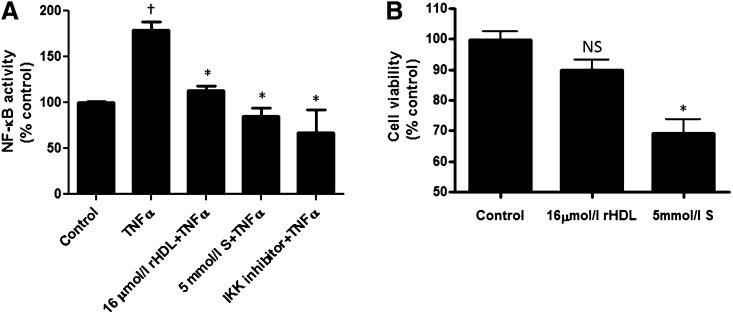
Inhibition of TNFα-induced NF-κB activity by rHDLs versus salicylate. A: HuH-7 cells were transfected with an NF-κB-luciferase reporter vector then preincubated for 16 h with PBS (control), rHDLs (final apoAI concentration 16 μmol/l or 0.45 mg/ml), sodium salicylate (S) (5 mmol/l), or the IKK inhibitor (wedelolactone) prior to stimulation with TNFα for 5 h. The cells were harvested, lysed, and the cell lysates were assayed for luciferase activity. Results are expressed as mean ± SEM (n = 5). †*P* < 0.05 versus control; *P < 0.05 versus TNFα. B: HuH-7 cells were preincubated for 16 h with PBS (control), rHDLs (final apoAI concentration 16 μmol/l or 0.45 mg/ml), or salicylate (S) (5 mmol/l) for 40 h. Cell viability was then measured by the lactate dehydrogenase assay. Results are expressed as mean ± SEM (n = 5). *P < 0.05 versus control. NS, not significant.

Sodium salicylate suppresses hepatic NF-κB activity and improves insulin sensitivity in C57BL/6 mice (22). We found that pretreatment of the cells with rHDLs is as effective as 5 mmol/l of sodium salicylate in suppressing TNFα-activated NF-κB activation. HuH-7 cells were transfected with the NF-κB-luciferase reporter vector and then exposed to rHDLs (final apoAI concentration, 16 μmol/l or 0.45 mg/ml) or 5 mmol/l sodium salicylate for 16 h prior to a 5 h TNFα activation. **Figure 7A** shows that rHDLs suppressed TNFα-induced NF-κB activation to levels similar to those observed with both 5 mmol/l sodium salicylate and the IKK inhibitor, wedelolactone. Additionally, unlike 5 mmol/l sodium salicylate, rHDL treatment had no effect on cell viability (Fig. 7B).

### rHDLs inhibit NF-κB target gene expression

NF-κB is a central mediator of the inflammatory response in many cell types (20, 21). Using a NF-κB target gene macroarray, it was determined that pretreatment with rHDLs decreased the expression of a number of target genes, including cytokines/chemokines (*IL8*, *MCP1*, *PTX3*, *GRO1*, *SAA1*), a proteinase inhibitor (*A1AT*), cell differentiation and proliferation associated genes (*GAL3*, *MAD3*, *TP53*, *PRG1*), reactive oxygen stress associated genes (*MNSOD*, *GSTP1*), and cell adhesion molecules (*LAMB2*) (supplementary Fig. V). The macroarray results were confirmed by RT-qPCR (**Table 2**).

**TABLE 2.** Confirmation of NF-κB-targeted genes in HuH-7 cells by RT-qPCR

Gene Assignment	GenBank	Fold Change TNFα + HDL/TNFα	Function
*A1AT*, α-1 protease inhibitor	NM_001002236	0.78 ± 0.04*^a^*	Inhibits elastase, plasmin, thrombin, trypsin, and plasminogen activator
*GAL3*, galactose-specific lectin	NR_003225	0.64 ± 0.03*^b^*	Involvement in G protein-coupled receptor protein signaling pathway, cell differentiation, and extracellular matrix organization
*GRO1*, chemokine (C-X-C motif) ligand 1	NM_001511	0.53 ± 0.04*^b^*	Chemokine, inflammatory response
*GSTP1*, glutathione S-transferase pi 1	NM_000852	0.88 ± 0.04*^a^*	Important role in detoxification by conjugation of reduced glutathione to a wide number of exogenous and endogenous hydrophobic electrophiles
*IGFBP2*, insulin-like growth factor binding protein 2	NM_000597	0.69 ± 0.08*^a^*	Prolongs the half-life of IGFs, stimulates or inhibits the growth promoting effects of Igs on cell culture
*IL8*, interleukin 8	NM_000584	0.73 ± 0.05*^a^*	Chemokine, major mediator of the inflammatory response
*LAMB2*, insulin-like growth factor binding protein 2	NM_002292	0.74 ± 0.06*^b^*	Major noncollagenous constituent of basement membranes involved in cell adhesion
*MAD3*, max-associated protein 3	NM_031300	0.90 ± 0.03*^b^*	Transcriptional regulator and part of the Myc superfamily. Binds with MAX and suppresses MYC-dependent cell transformation
*MCP1*, monocyte chemoattractant protein 1	NM_002982	0.63 ± 0.08*^a^*	Chemokine, inflammatory response
*MNSOD*, manganese-containing superoxide dismutase	NM_001024465	0.65 ± 0.03*^b^*	Clears toxic oxidative radicals produced within cells
*TP53*, tumor suppressor p53	NM_000546	0.89 ± 0.02*^b^*	Regulates target genes that induce cell cycle arrest, apoptosis, senescence, DNA repair, or changes in metabolism. High levels are associated with transformation and malignancy
*PRG1*, p53-responsive gene 1	NM_003897	0.65 ± 0.06*^b^*	
*PTX3*, pentraxin 3	NM_002852	0.80 ± 0.04*^b^*	Role in regulation of inflammation
*SAA1*, serum amyloid A1	NM_000331	0.71 ± 0.03*^b^*	Major acute inflammatory reactant, associated with HDL

a *P* < 0.05.

b *P* < 0.01.

## DISCUSSION

High-fat feeding of mice induces systemic and hepatic inflammation, increases hepatic NF-κB activation, impairs glucose tolerance, and induces insulin resistance (4, 23). The present study shows that intravenous infusion of apoAI into these animals reversed all of these changes. In vitro, TNFα-induced inflammation of hepatocytes resulted in increased IKK activity, increased phosphorylation of IκBα, and increased activation of NF-κB. Exposure of these activated hepatocytes to apoAI (as a component of rHDLs) reversed all of these changes.

The present study is the first to show that administration of apoAI is able to improve glucose tolerance and insulin sensitivity in HFD-fed C57BL/6 mice. The proposed mechanism involves anti-inflammatory effects via suppression of hepatic NF-κB activation. This mechanism is similar to that described for sodium salicylate in protecting against hepatic inflammation, and subsequently, the development of insulin resistance (4). Insulin resistance remains hard to treat, as to date there are few targeted medicines. We have previously demonstrated that rHDLs (with apoAI as the main protein) have strong anti-inflammatory effects in endothelial cells, a key cell type in atherosclerosis (12). Given that preparations of HDLs are currently in clinical development as anti-atherogenic agents, a logical next step will be to test the ability of these preparations to protect against the hepatic inflammation and insulin resistance that accompanies nonalcoholic fatty liver.

In this study we showed that apoAI treatment, either independently or as part of a reconstituted HDL particle, suppressed a number of inflammatory, oxidant, and apoptotic stimuli via signaling through the canonical NF-κB pathway. In previous studies, we have shown that administration of apoAI at a dosage of 8 mg/kg does not lead to a significant increase in the circulating HDL level (24) and, as such, the molecular mechanism activated in vivo by an apoAI injection to mediate the anti-inflammatory effects on hepatic NF-κB activation remains to be determined, although it may involve upregulation of DHCR24 and other cellular protective enzymes. We have previously shown that apoAI, as a component of discoidal rHDLs, suppressed NF-κB activation through increased expression of DHCR24 (a cellular protective enzyme) levels, and that this enzyme is at least in part responsible for mediating the anti-inflammatory effects of rHDLs in human coronary aortic endothelial cells (12). However, in the current study, there was no increase in DHCR24 expression, at least at the mRNA level, in the liver of apoAI-treated mice; so it is likely that another mechanism is responsible for HDLs’ protection against NF-κB activation. One of the major protective properties of HDLs revolves around their ability to promote cholesterol efflux. It has been shown in macrophages, endothelial cells, and recently adipocytes (25–27) that the anti-inflammatory effects mediated by HDLs are due, at least in part, to cholesterol efflux. However, in this study, we found that modulation of membrane cholesterol using methyl-β-cyclodextrin (depletes membrane cholesterol) or HDLs had no effect on NF-κB activation, suggesting that cholesterol efflux does not have a key role in the anti-inflammatory effects of HDLs in hepatocytes. The underlying mechanism now needs to be interrogated further for a complete understanding.

We have also shown that 2 and 4 weeks of apoAI treatment decreased expression of the gluconeogenesis-associated PEPCK and G6Pase, an effect that correlated with an improved response to the pyruvate challenge. Thus, in addition to the effects on insulin resistance, apoAI may decrease glucose output from the liver via suppression of gluconeogenesis, most likely through effects on NF-κB.

Improvement in systemic insulin resistance and glucose homeostasis as a result of apoAI treatment may be an additive consequence of effects on multiple organs including the liver, pancreas, adipose tissue, and skeletal muscle. We have previously reported that apoAI treatment of cultured pancreatic β-cells increased both the synthesis and secretion of insulin in the setting of both high and low glucose concentrations (28). In keeping with this finding, apoAI as a component of rHDLs has also been shown to reduce plasma glucose levels in patients with type 2 diabetes, associated with an rHDL-mediated increase in plasma insulin (28). ApoAI rHDLs were shown to increase AMP-activated protein kinase activity in skeletal muscle in type 2 diabetes patients (29), and that this was a key pathway to glucose reduction in these patients as increased AMP-activated protein kinase induces increased glucose uptake by skeletal muscle cells. In a study of apoAI-transgenic mice, rather than apoAI treatment, it was shown that adipose tissue inflammation induced by a high-fat high-sucrose diet was ameliorated, relative to wild-type mice, in keeping with reports that HDL and apoAI have anti-inflammatory effects on adipocytes (30). A direct anti-obesity effect on apoAI and its mimetic peptide has also been recently reported whereby HDL treatment increased adipose tissue expenditure through attainment of brown adipocyte phenotype in white adipose tissue (31). ApoAI possesses an anti-obesity effect associated with an increase of energy expenditure and upregulation of UCP1 in brown fat. We now show that apoAI decreases liver inflammation by suppressing NF-κB activation. It is likely that the profound ability of apoAI as a component of rHDLs to improve insulin resistance is a combined effect on the liver, adipose tissue, pancreas, and skeletal muscle. We suspect that apoAI-induced suppression of hepatic NF-κB activation is central to the overall protective effect of apoAI on insulin resistance, a suggestion consistent with the observations of Shoelson and colleagues, who demonstrated that NF-κB activation alone in the liver is sufficient to drive the onset of insulin resistance in mice (4).

A limitation of this study is that it was completed in a mouse model and therefore may not reflect what is clinically relevant in human subjects. However, recent findings in humans show that HDL-raising interventions are associated with an increase in insulin sensitivity in patients with type 2 diabetes (32). More direct evidence of HDL improving insulin resistance in humans is provided in a study showing infusion of rHDL particles increases plasma insulin levels, and decreases plasma glucose levels in type 2 diabetic subjects (29). Moreover, salsalate, a nonacetylated salicylate, was used to treat patients with type 2 diabetes and in this study reduced HbA1c levels (33), thereby providing evidence for the hypothesis that an anti-inflammatory approach could be used to treat insulin resistance and subsequent type 2 diabetes. However, to date, the mechanisms by which HDLs improve insulin sensitivity in humans, and whether it involves anti-inflammatory effects as we have now demonstrated in C57BL/6 mice, remains to be investigated.

In summary, our findings that apoAI can improve insulin sensitivity through anti-inflammatory effects in hepatocytes may lead to novel approaches for the treatment of insulin resistance. We have shown that apoAI, as a component of rHDLs, potently suppresses the key mediator of inflammation, NF-κB, and in doing so can decrease the hepatic inflammation that underlies the onset of insulin resistance. HDL cholesterol levels are often reduced as a consequence of insulin resistance (13, 34). The results from this study, therefore, have important therapeutic implications, whereby raising HDL levels has the potential to protect against hepatic inflammation, and subsequently, insulin resistance.

## Supplementary Material

Supplemental Data
