# Supplementary material for: High density lipoproteins improve insulin sensitivity in high-fat diet-fed mice by suppressing hepatic inflammation
Source: J Lipid Res. 2014 Mar;55(3):421–30. doi: 10.1194/jlr.M043281 (PMC3934727; doi:10.1194/jlr.M043281)
Supplement: Supplemental Data [file supp_55_3_421__index.html]

High density lipoproteins improve insulin sensitivity in high fat diet fed mice by suppressing hepatic inflammation — High density lipoproteins improve insulin sensitivity in high-fat diet-fed mice by suppressing hepatic inflammation — Supplemental Data 

# High density lipoproteins improve insulin sensitivity in high-fat diet-fed mice by suppressing hepatic inflammation

## Supplemental Data

**Files in this Data Supplement:**

- ESM Fig. I - Insulin resistance in C57BL/6 mice treated with apoAI
- ESM Fig. II - Fasting blood glucose and serum insulin levels
- ESM Fig. III - Circulating triglyceride and hepatic neutral lipid (triglyceride and cholesterol esters) levels.
- Supplemental Data - ApoAI does not affect hepatic expression of DHCR24 mRNA levels.
- ESM Fig. V - AI-rHDLs suppress TNF&#x26;#61537;-activated NF-&#x26;#61547;B target gene expression in HuH-7 cells.
- ESM Table I - Quantitative real-time PCR primer sequences.
