# Supplementary figures and images for: High density lipoproteins improve insulin sensitivity in high-fat diet-fed mice by suppressing hepatic inflammation
Source: J Lipid Res. 2014 Mar;55(3):421–30. doi: 10.1194/jlr.M043281 (PMC3934727; doi:10.1194/jlr.M043281)

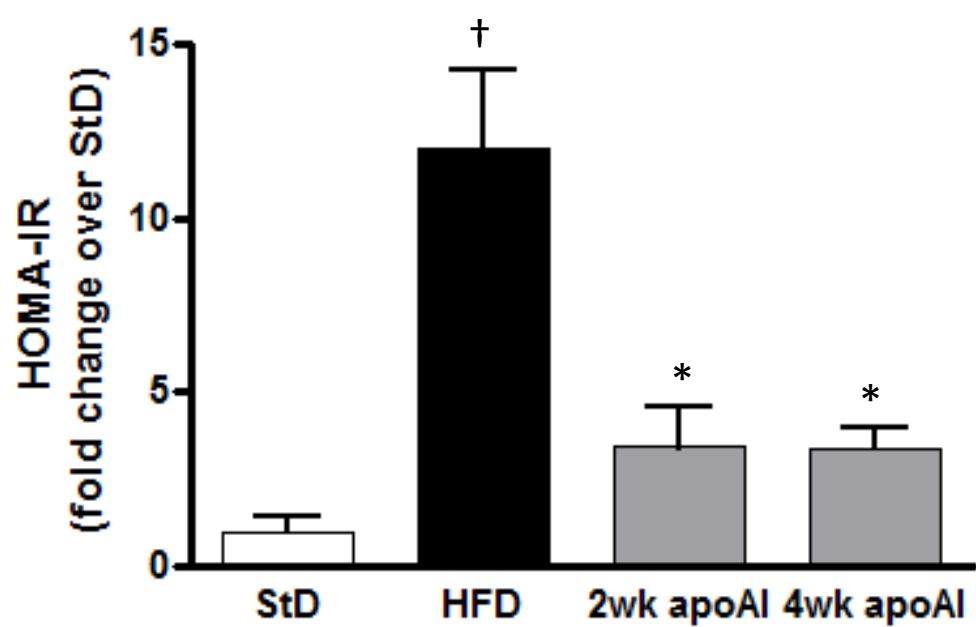

Supplement: Supplemental Data [file supp_M043281_jlr.M043281-1.pdf]

**A**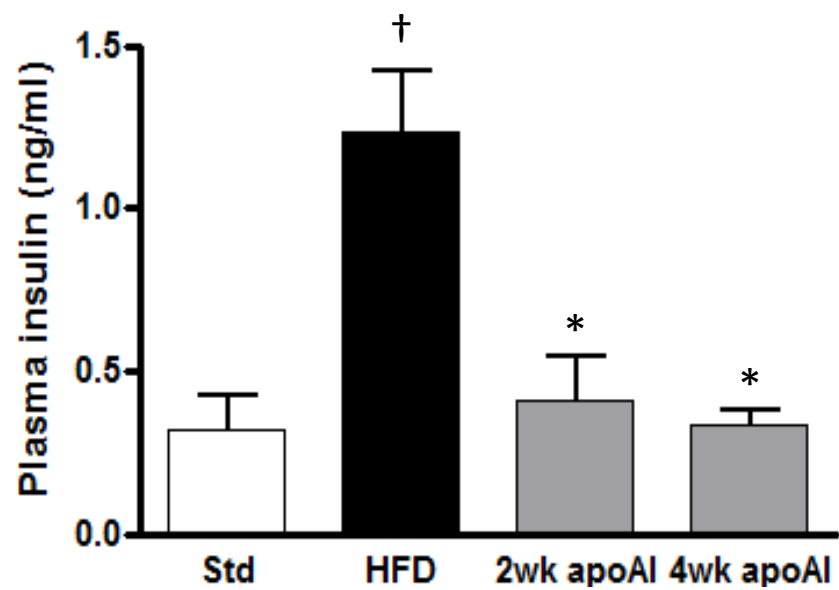**B**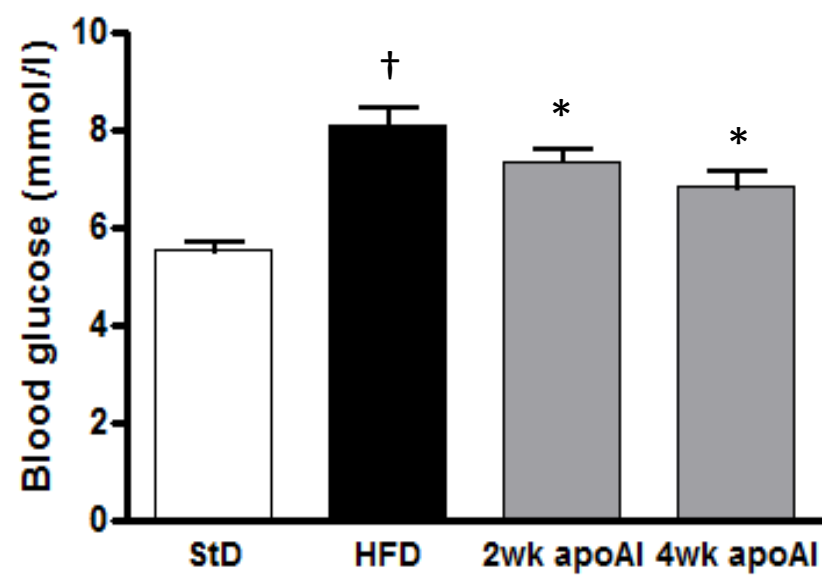

Supplement: Supplemental Data [file supp_M043281_jlr.M043281-2.pdf]

**A**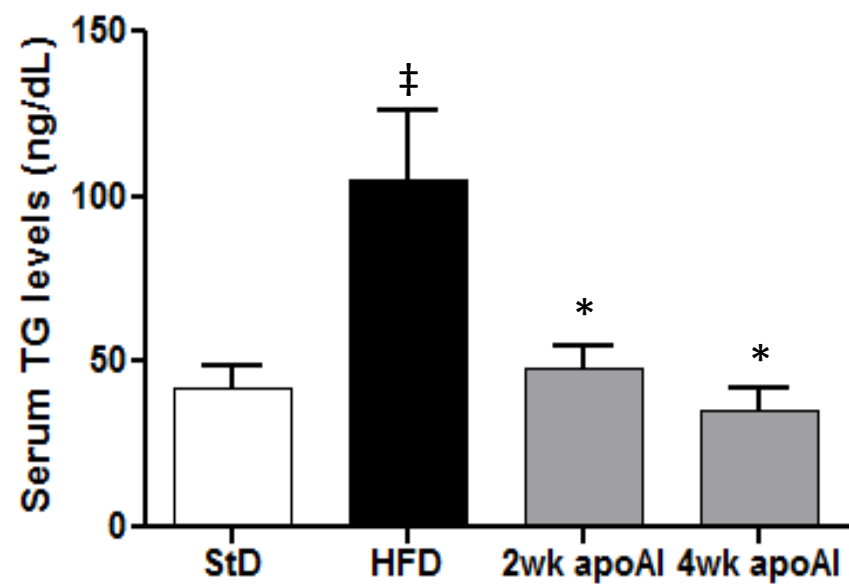**B**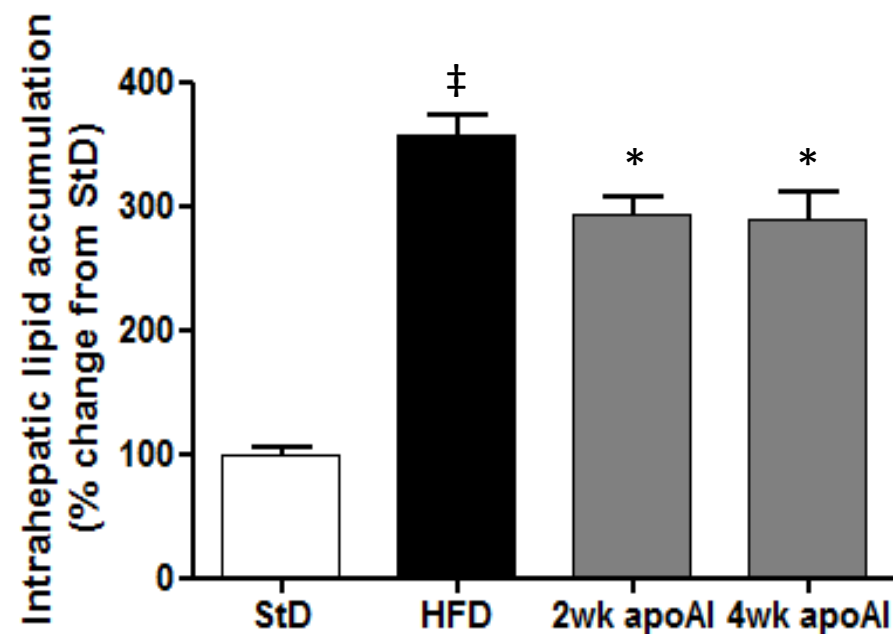

Supplement: Supplemental Data [file supp_M043281_jlr.M043281-3.pdf]

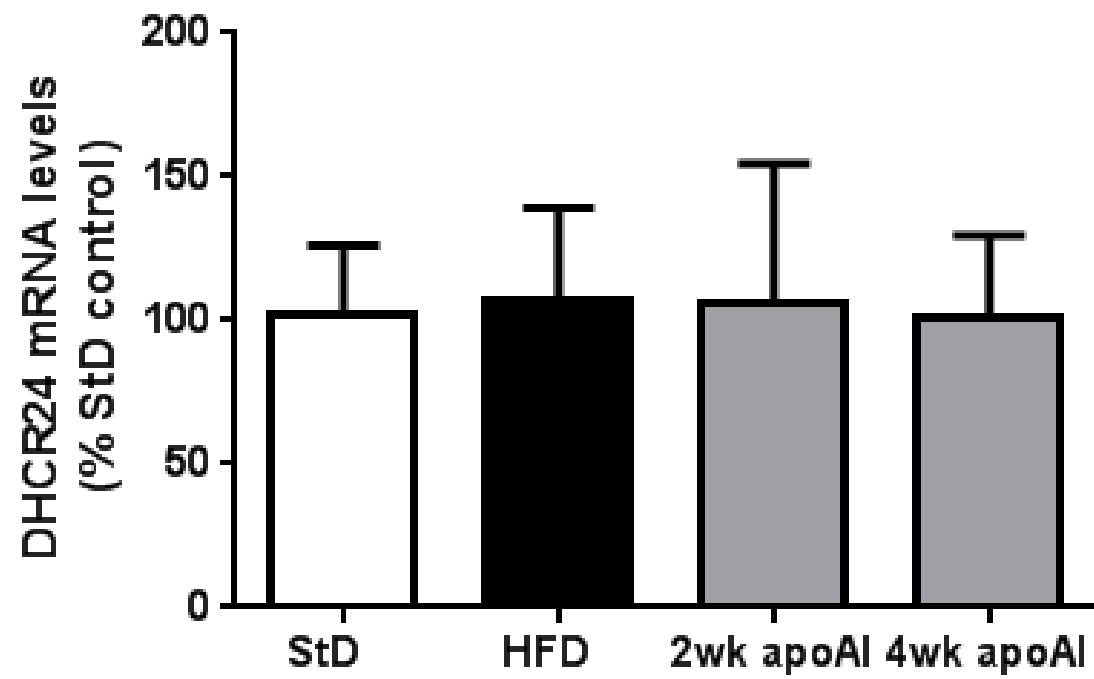

Supplement: Supplemental Data [file supp_M043281_jlr.M043281-4.pdf]
