# Supplementary material for: High density lipoproteins improve insulin sensitivity in high-fat diet-fed mice by suppressing hepatic inflammation
Source: J Lipid Res. 2014 Mar;55(3):421–30. doi: 10.1194/jlr.M043281 (PMC3934727; doi:10.1194/jlr.M043281)
Supplement: Supplemental Data [file supp_M043281_jlr.M043281-5.pdf]

A

PBS + TNFα   rHDL + TNFα

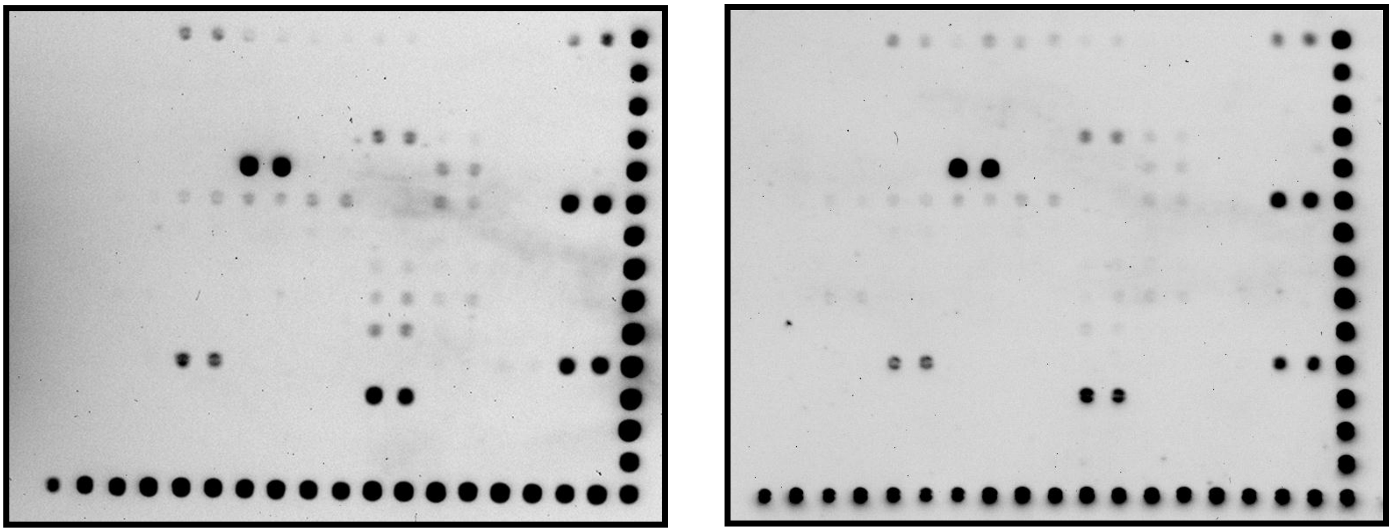

B

A	ALOX12	ALOX12	ADORA1	ADORA1	A1AT	A1AT	A20	A20	a1 acid GP	a1 acid GP	AGT	AGT	APOC3	APOC3	HLA-G1	HLA-G1	ACTB	ACTB		A
B	CD80	CD80	BCL-x1	BCL-x1	BCL2A1	BCL2A1	BGN	BGN	IFNb	IFNb	BDKRB1	BDKRB1	BRL1	BRL1	CCR5	CCR5				B
C	CD23	CD23	CD48	CD48	CD69	CD69	CD95	CD95	UGT8	UGT8	c-myb	c-myb	c-myc	c-myc	MMP1	MMP1				C
D	FB	FB	COX-2	COX-2	c-rel	c-rel	SCYB11	SCYB11	CYC-D1	CYC-D1	CCND3	CCND3	DDH1	DDH1	ELAM-1	ELAM-1				D
E	SCYA11	SCYA11	F8	F8	Fas-L	Fas-L	FTH	FTH	GAD65	GAD65	Gal1-R	Gal1-R	GAL-3	GAL-3	CSF3	CSF3				E
F	CSF2	CSF2	GRO1	GRO1	GSTP1	GSTP1	HMG-14	HMG-14	HMOX1	HMOX1	HAS1	HAS1	ICAM-1	ICAM-1	IFNg	IFNg	GAPDH	GAPDH		F
G	IGFBP1	IGFBP1	IGFBP-2	IGFBP-2	MAD-3	MAD-3	IL10	IL10	IL11	IL11	IL12	IL12	IL15	IL15	IL1-a	IL1-a				G
H	IL1B	IL1B	IL1RN	IL1RN	IL2	IL2	IL2-Ra	IL2-Ra	IL6	IL6	IL8	IL8	IL9	IL9	NOS	NOS				H
I	IRF1	IRF1	IRF-2	IRF-2	JUN-B	JUN-B	LAMB2	LAMB2	Lox-1	Lox-1	LYZ	LYZ	MAdCAM-1	MAdCAM-1	MCP-1	MCP-1				I
J	CSF-1	CSF-1	MDR-1	MDR-1	MIP2g	MIP2g	MMP-3	MMP-3	MMP9	MMP9	Mn-SOD	Mn-SOD	MTS1	MTS1	NPYY1	NPYY1				J
K	NFKB1	NFKB1	NFKB2	NFKB2	PRG1	PRG1	p53	p53	PAFR1	PAFR1	PAX8	PAX8	PDGF-B	PDGF-B	PTX3	PTX3	UBC	UBC		K
L	TAP1	TAP1	PENK	PENK	LMP-2	LMP-2	CD62	CD62	PTGIS	PTGIS	AGER	AGER	RANTES	RANTES	SAA	SAA				L
M	TCRB	TCRB	TNC	TNC	THBS2	THBS2	TNF	TNF	TNFb	TNFb	TNF-R	TNF-R	TGM1	TGM1	UPAR	UPAR				M
N	VCAM-1	VCAM-1	VEGFC	VEGFC	VIM	VIM	WT1	WT1	TNSFS5	TNSFS5	MSX1	MSX1	AhRR	AhRR						N
O																				O
	1	2	3	4	5	6	7	8	9	10	11	12	13	14	15	16	17	18		
