# Supplementary material for: High density lipoproteins improve insulin sensitivity in high-fat diet-fed mice by suppressing hepatic inflammation
Source: J Lipid Res. 2014 Mar;55(3):421–30. doi: 10.1194/jlr.M043281 (PMC3934727; doi:10.1194/jlr.M043281)
Supplement: Supplemental Data [file supp_M043281_jlr.M043281-6.pdf]

Gene	Accession no.	Forward	Reverse
A1AT	NM_001002236	CAATGCCACCGCCATCTTCTTC	CAGTTGACCCAGGACGCTCTT
GAL3	NR_003225	CCTTCCACTTTAACCCACGCTTC	ACCGACTGTCTTTCTTCCCTTCC
GRO1	NM_001511	TGCTGCTCCTGCTCCTGGTA	GTGTGGCTATGACTTCGGTTTGG
GSTP1	NM_000852	TCGCCGCCGCAGTCTTC	GTCACCACCTCCTCCTTCCAG
IGFBP2	NM_000597	GGGAGTGCTGGTGTGTGAAC	CGGCTGGCTGCGGTCTA
1L8	NM_000584	CGGAAGGAACCATCTCACTGT	GGTCCACTCTCAATCACTCTCA
LAMB2	NM_002292	TGTGATTGTGACTCTCGTGGAAT	CAGGGATGGCAGGCAGGAA
MAD3	NM_031300	GCTGGAGGTGGATGTGGAGAG	GGCACGAGTAGAGGGCAGAG
MCP1	NM_002982	CAA TCA ATG CCC CAG TCA C	GAT TCT TGG GTT GTG GGA GTG
MNSOD	NM_001024465	CCTAACGGTGGTGGAGAAC	AACCTGAGCCTTGGACAC
TP53	NM_000546	GCATCTACAAGCAGTCACAGC	TCCACACGCAAATTTCCTTCC
PRG1	NM_003897	GCATCCTCCAGCATCTCAACTC	CTACCTCGCAGCCACCCTAAA
PTX3	NM_002852	GTGGGTGGTGGCTTTGATGAAA	GATGTGACAAGACTCTGCTCCTC
B2M	BC064910.1	CATCCAGCGTACTCCAAAGA	GACAAGTCTGAATGCTCCAC
SAA1	NM_000331	CCAATCACTTCCGACCTGCTG	GCTTTGTATCCCTGCCCTGAG
CHREBP	NM_021455.4	CTG GGG ACC TAA ACA GGA GC	GAA GCC ACC CTA TAG CTC CC
PEPCK	NM_011044.2	GGTGTTTACTGGGAAGGCATC	CAATAATGGGGCACTGGCTG
G6P	NM_008061.3	CATGGGCGCAGCAGGTGTATACT	CAAGGTAGATCCGGGACAGACAG
TNFα	BC137720.1	CTGTGAAGGGAATGGGTGTT	CTCCCTTTGCAGAACTCAGG
IL6	DQ788722.1	TGTGCAATGGCAATTCTGAT	GGAAATTGGGGTAGGAAGGA
IFNγ	BC119065.1	GAAAAGGAGTCGCTGCTGAT	CGCAATCACAGTCTTGGCTA
IL1β	M15131.1	GCTCAGGGTCACAAGAAACC	CATCAAAGCAATGTGCTGGT
CD68	BC021637.1	CCAATTCAGGGTGGAAGAAA	CTCGGGCTCTGATGTAGGTC
F4/80	X93328.1	CTCCAAGCCTATTATCTATACC	CTTCCACAATCTCACAGC
SREBP1	NM_011480.3	TTTCCTTAACGTGGGCCTAGTC	TGTCTTCGATGTCGTTCAAAACC
DHCR24	NM_053272.2	GAAGTCAACGCAAGCCTCTCC	ACATCGCCACACCCATCCC
TBP	U63933.1	GGCCTCTCAGAAGCATCACTA	GCCAAGCCCTGAGCATAA
